# Challenges Using Technology in a Health Coaching Intervention, Early Lessons Learned

**DOI:** 10.1093/geroni/igaa057.2255

**Published:** 2020-12-16

**Authors:** Barbara Riegel, Karen Hirschman, Gladys Thomas, Brooke Shepard, Tracie Walser, Michael Stawnychy, Kathryn Bowles, Lydia Garcia

**Affiliations:** 1 University of Pennsylvania, Philadelphia, Pennsylvania, United States; 2 NewCourtland Center for Transitions and Health, Philadelphia, Pennsylvania, United States; 3 University Of Pennsylvania School of Nursing, Philadelphia, Pennsylvania, United States; 4 University of Pennsylvania, Lincoln, United States; 5 University of Pennsylvania School of Nursing, Philadelphia, Pennsylvania, United States; 6 University of Pennsylvania, Oakland, California, United States

## Abstract

Technology provides opportunities to engage with those who are too busy, overwhelmed, or distant for face-to-face interventions, such as lay caregivers of adults with heart failure. While testing the efficacy of virtual health coaching for caregivers on stress and self-care we have encountered challenges in implementing the intervention and learned early lessons. Caregivers (n=250) enrolled into a randomized controlled trial receive a Samsung Galaxy tablet with Internet access. Half receive health information by tablet (control) and half receive 10 live virtual health coaching sessions plus health information all by tablet (intervention). Tablets are configured to allow access to preselected websites and a Vidyo conference room for the intervention group. Completing the first 6 months of enrollment, 36 caregivers have enrolled (34% black, all female, 74% spouses, mean age 56.4 years, 40% employed full-time, mean 9.3 hours spent caregiving daily), with 18 randomized to intervention. Only 2 of the 79 assessed for eligibility reported discomfort with technology. Yet, one early challenge is difficulty connecting with the health coach via Vidyo. Another is the uncertain nature of the patient’s condition, which frequently precludes the caregivers’ attendance at prescheduled sessions. Although tablets were anticipated to facilitate the intervention, in some cases, these two challenges interact to further accentuate caregiver stress. Alternatives such as FaceTime and plain old telephone contact suffice. We found that flexibility in intervention delivery is essential for such a real-world intervention. Contributing factors and implications of these challenges for future caregiving research and practice will be discussed.

